# A neutral comparative analysis of additive, multiplicative, and mixed quantitative randomized response models

**DOI:** 10.1371/journal.pone.0284995

**Published:** 2023-04-26

**Authors:** Muhammad Azeem, Sidra Ali

**Affiliations:** Department of Statistics, University of Malakand, Lower Dir, KP, Pakistan; Universiti Teknologi Malaysia, MALAYSIA

## Abstract

In survey sampling, the randomized response technique is a useful tool to collect reliable data in many fields including sociology, education, economics, and psychology etc. Over the past few decades, many variants of quantitative randomized response models have been developed by researchers. The existing literature on randomized response models lacks a neutral comparative study of different models to help the practitioners choose the appropriate model for a given practical problem. In most of the existing studies, the authors tend to show only the favorable results by hiding the cases where their suggested models are inferior to the existing models. This approach often leads to biased comparisons which may badly misguide the practitioners when choosing a randomized response model for a practical problem at hand. This paper attempts a neutral comparison of six existing quantitative randomized response models using separate as well as joint measures of respondent-privacy and model-efficiency. The findings suggest that one model may perform better than the other model in terms of efficiency but may perform worse when other metrics of model quality are taken into account. The current study guides practitioners in choosing the right model for a given problem under a particular situation.

## 1. Introduction

Survey researchers often face refusals and false responses by respondents while collecting data on sensitive variables. A few examples of sensitive variables are: cheating in examination, illegal income, expenditure on luxury items, the amount of tax payable, and the number of cigarettes used per day etc. In order to deal with non-response on questions regarding sensitive characteristics, a useful procedure, popularly known as the randomized response technique, was suggested by Warner [1]. The original randomized response technique was designed for practical situations where the researcher collects data on binary-type qualitative variables. Warner [2] extended the original qualitative technique to the case of quantitative variables by introducing an additive-type scrambling. Motivated by Warner [2], a new variant of the quantitative scrambling techniques was suggested by Eichhorn and Hayre [3] by using a multiplicative-type scrambling variable.

Gupta et al. [4] devised a randomized strategy where the respondents are offered the choice to report either the true or a scrambled response. If a respondent opts for the scrambled response, he/she has to use an additive-type scrambling procedure to report the response. Later on, a multiplicative version of the Gupta et al. [4] procedure was suggested by Bar-Lev et al. [5]. Gjestvang and Singh [6] introduced an additive-type scrambling procedure for data collection on quantitative sensitive variables. Diana and Perri [7] suggested a quantitative randomized technique by utilizing both additive and multiplicative-type scrambling noises. Al-Sobhi et al. [8] introduced a new quantitative technique by using additive-subtractive scrambling noise. Gupta et al. [9] presented a measure for evaluation of randomized response models by quantifying the respondents’ privacy and efficiency as a single number. Narjis and Shabbir [10] suggested an efficient variant of the Gjestvang and Singh [6] technique for data collection on quantitative sensitive variables. Khalil et al. [11] analyzed the influence of measurement errors on the mean estimator of the sensitive quantitative variable. Recently, Gupta et al. [12] introduced a new quantitative randomized technique, showing the improvement over the Diana and Perri [7] technique with regard to the respondents’ privacy as well as the efficiency of model. In addition to the above studies, various aspects of the randomized response techniques have been analyzed by Yan et al. [13], Kalucha et al. [14], Young et al. [15], Zhang et al. [16], Murtaza et al. [17], Zapata et al. [18], and Saleem and Sanaullah [19] etc.

Chen et al. [20] presented the direct probability integral method to cope with Stochastic response and global dynamic analyses of structures with chaotic motion. Torkayesh et al. [21] presented a new method for minimizing air pollutants and to enhance environmental sustainability. Mondal et al. [22] analyzed the robustness of multilayer perceptron with regard to additive or multiplicative input noise. Silva et al. [23] recently studied a Bayesian analysis of the additive main effects and multiplicative interaction model utilizing three a priori distributions. Akgun et al. [24] studied multi-objective optimization for the rating of carbon-based additives in phase change materials using different criteria for evaluation.

Recently, Singh et al. [25] developed two new quantitative randomized response models which were shown to be better than the existing models in terms of efficiency as well as privacy protection level. In another study, Singh et al. [26] utilized Poisson distribution to develop a three-stage randomized response model which helps in estimating the mean number of persons having a sensitive attribute. The Singh et al. [26] model improved the efficiency of the existing models.

The research studies mentioned above have presented many variants of randomized response models. Some of these existing models utilize additive scrambling variables whereas others use multiplicative scrambling or mixed scrambling where both additive and multiplicative variables have been used by researchers. However, to our knowledge, no attempt has been made to conduct a detailed comparative analysis of the different versions of the existing randomized response models. The present study compares six existing randomized response models using three measures of model-quality: (i) model efficiency, (ii) measure of respondent-privacy, and (iii) joint measure of model-efficiency and respondent-privacy.

## 2. Selected existing models for comparative analysis

In the current study, we have chosen six existing quantitative randomized response models for comparative analysis. Out of the six selected models for comparison, two models are based on scrambled responses with no option for true response. The next two of the selected six models are optional models, including one additive and one multiplicative model. Finally, the last two of the selected six models are mixed models, that is, they use both additive and multiplicative scrambling noise. Before proceeding to comparative analysis, we introduce the notations used for the variables and their parameters, along with some distributional assumptions under which the comparative study is carried out.

Let the population under study contains *N* units and let a simple random sample of size *n* units is obtained with replacement. Let the quantitative sensitive variable under study be denoted by *Y*, and let the additive scrambling variable be denoted by *S*. We further assume that *E*(*Y*_*i*_) = *μ*_*Y*_, *E*(*S*) = *θ*, $V(Y_{i})=\sigma_{Y}^{2}$, $V(S)=\sigma_{S}^{2}$, where $\sigma_{Y}^{2}$ and $\sigma_{S}^{2}$ are the variances of the variable *Y* and *S*, respectively, for the population data. Further, let *μ*_*Y*_ and *θ* be the population means of the variable *Y* and *S*, respectively. Likewise, let *T* be a multiplicative-type scrambling variable, such that *E*(*T*) =1, and $Var(T)=\sigma_{T}^{2}$. It is also assumed that all of the three variables work independently of each other. In this section, some existing quantitative scrambling techniques are presented.

### 2.1 Warner’s [2] model

The additive model suggested by Warner [2] is as follows:

Z=Y+S, (1)

where *Z* is the reported response. An unbiased mean estimator of *Y* based on Warner’s [2] model is given as:

$${\hat{\mu }}_{W}=\frac{1}{n}\sum_{i=1}^{n}Z_{i}.$$ (2)

The variance of ${\hat{\mu }}_{W}$ is given as:

$$Var({\hat{\mu }}_{W})=\frac{1}{n}(\sigma_{Y}^{2}+\sigma_{S}^{2}).$$ (3)

### 2.2 Eichhorn and Hayre [3] model

The responses reported by the respondents under the Eichhorn and Hayre [3] model, are as follows:

Z=TY. (4)

An unbiased estimator of *μ*_*Y*_ under the Eichhorn and Hayre [3] technique is:

$${\hat{\mu }}_{EH}=\frac{1}{n}\sum_{i=1}^{n}Z_{i}.$$ (5)

The variance of ${\hat{\mu }}_{EH}$ is given as:

$$Var({\hat{\mu }}_{EH})=\frac{\sigma_{Y}^{2}}{n}+\frac{\sigma_{T}^{2}(\sigma_{Y}^{2}+\mu_{Y}^{2})}{n}.$$ (6)

### 2.3 Gupta et al. [4] model

The reported responses under the Gupta et al. [4] model are given as:

$$Z=\{\begin{array}{ll} Y, & \mathrm{with}\;\mathrm{probability}\;p\\ Y+S, & \mathrm{with}\;\mathrm{probability}\;1-p. \end{array}$$ (7)

The mean estimator under the Gupta et al. [4] technique is given by:

$${\hat{\mu }}_{G1}=\frac{1}{n}\sum_{i=1}^{n}Z_{i},$$ (8)

where *Z* is defined in Eq (7). The variance of the mean estimator is as follows:

$$Var({\hat{\mu }}_{G1})=\frac{1}{n}[\sigma_{Y}^{2}+(1-p)\sigma_{S}^{2}].$$ (9)

### 2.4 Bar-Lev et al. [5] model

The responses reported by the respondents under the Bar-Lev et al. [5] technique, are as follows:

$$Z=\{\begin{array}{ll} Y, & \mathrm{with}\;\mathrm{probability}\;p\\ TY, & \mathrm{with}\;\mathrm{probability}\;1-p. \end{array}$$ (10)

The mean estimator under the Bar-Lev et al. [5] technique is given by:

$${\hat{\mu }}_{B}=\frac{1}{n}\sum_{i=1}^{n}Z_{i},$$ (11)

where *Z* is defined in Eq (10). The variance of the mean estimator is as follows:

$$Var({\hat{\mu }}_{B})=\frac{1}{n}[\sigma_{Y}^{2}+(1-p)\sigma_{T}^{2}(\sigma_{Y}^{2}+\mu_{Y}^{2})].$$ (12)

### 2.5 Murtaza et al. [17] model

The reported responses under the Murtaza et al. [17] model, are given as:

$$Z=\{\begin{array}{ll} Y, & \mathrm{with}\;\mathrm{probability}\;p\\ TY+\alpha S, & \mathrm{with}\;\mathrm{probability}\;1-p, \end{array}$$ (13)

where *α* is a constant. An unbiased mean estimator of the sensitive variable based on Murtaza et al. [17] model is given as:

$${\hat{\mu }}_{M}=\frac{1}{n}\sum_{i=1}^{n}Z_{i}.$$ (14)

The Murtaza et al. [17] model is based on correlated scrambling variables. In order to make the comparison feasible, the assumption of uncorrelated variables is used, as the other models selected for comparison also use uncorrelated scrambling variables. The variance of ${\hat{\mu }}_{M}$ is given by:

$$Var({\hat{\mu }}_{M})=\frac{1}{n}[(1-p)\sigma_{T}^{2}(\sigma_{Y}^{2}+\mu_{Y}^{2})+\sigma_{Y}^{2}+\alpha^{2}\sigma_{S}^{2}].$$ (15)

### 2.6 Gupta et al. [12] model

Gupta et al. [12] introduced the following optional scrambling model:

$$Z=\{\begin{array}{ll} Y & \mathrm{with}\;\mathrm{probability}\;p\\ Y+S & \mathrm{with}\;\mathrm{probability}\;(1-p)A\\ TY+S & \mathrm{with}\;\mathrm{probability}\;(1-p)(1-A), \end{array}$$ (16)

where *A* is a constant, 0 < *A* < 1. An unbiased mean estimator on the basis of the Gupta et al. [12] model, is given by:

$${\hat{\mu }}_{G}=\frac{1}{n}\sum_{i=1}^{n}Z_{i}.$$ (17)

The sampling variance of ${\hat{\mu }}_{G}$ is as follows:

$$Var({\hat{\mu }}_{G})=\frac{1}{n}[(1-p)(1-A)\sigma_{T}^{2}(\sigma_{Y}^{2}+\mu_{Y}^{2})+\sigma_{Y}^{2}+(1-p)\sigma_{S}^{2}].$$ (18)

## 3. Privacy and efficiency metrics

The Yan et al. [13] measure for quantifying the respondent-privacy is as follows:

$$\nabla =E{\left[Z-Y\right]}^{2}.$$ (19)

A higher the value of ∇ translates into a better level of respondents’ privacy provided by a given quantitative randomized response model.

The Gupta et al. [9] joint measure of efficiency and privacy-protection is as follows:

$$\delta =\frac{MSE}{\nabla }.$$ (20)

From Eq (20), one may clearly observe that lower values of *δ* are desirable.

For the Warner’s [2] model, the measure of privacy can be expressed as:

$$\nabla_{W}=E{\left[Y+S-Y\right]}^{2}=E(S^{2})=\sigma_{S}^{2}.$$ (21)

The joint measure of privacy and efficiency for the Warner’s [2] model is given as:

$$\delta_{W}=\frac{Var({\hat{\mu }}_{W})}{\nabla_{W}}=\frac{1}{n}(\frac{\sigma_{Y}^{2}+\sigma_{S}^{2}}{\sigma_{S}^{2}}).$$ (22)

For the Eichhorn and Hayre [3] quantitative technique, the measure of privacy can be obtained as:

$$\nabla_{EH}=E{\left[TY-Y\right]}^{2}=E(T^{2})E(Y^{2})+E(Y^{2})-2E(T)E(Y^{2}),$$

or,

$$\nabla_{EH}=\sigma_{T}^{2}(\sigma_{Y}^{2}+\mu_{Y}^{2}).$$ (23)

The joint measure of model-efficiency and respondent-privacy for the Eichhorn and Hayre [3] quantitative technique is given as:

$$\delta_{EH}=\frac{Var({\hat{\mu }}_{EH})}{\nabla_{EH}}=\frac{1}{n}[\frac{\sigma_{T}^{2}(\sigma_{Y}^{2}+\mu_{Y}^{2})+\sigma_{Y}^{2}}{\sigma_{T}^{2}(\sigma_{Y}^{2}+\mu_{Y}^{2})}].$$ (24)

The privacy level offered by the Gupta et al. [4] model is:

$$\nabla_{G1}=(1-p)\sigma_{S}^{2}.$$ (25)

The joint measure of model-efficiency and respondent-privacy for the Gupta et al. [4] quantitative technique is given as:

$$\delta_{G1}=\frac{Var({\hat{\mu }}_{G1})}{\nabla_{G1}}=\frac{1}{n}[\frac{\sigma_{Y}^{2}+(1-p)\sigma_{S}^{2}}{(1-p)\sigma_{S}^{2}}].$$ (26)

The measure of privacy for the Bar-Lev et al. [5] technique is:

$$\nabla_{B}=(1-p)\sigma_{T}^{2}(\sigma_{Y}^{2}+\mu_{Y}^{2}).$$ (27)

The joint measure of model-efficiency and respondent-privacy for the Bar-Lev et al. [5] quantitative technique is given as:

$$\delta_{B}=\frac{Var({\hat{\mu }}_{B})}{\nabla_{B}}=\frac{1}{n}[\frac{\sigma_{Y}^{2}+(1-p)\sigma_{T}^{2}(\sigma_{Y}^{2}+\mu_{Y}^{2})}{\left(1-p)\sigma_{T}^{2}(\sigma_{Y}^{2}+\mu_{Y}^{2}\right)}].$$ (28)

The measure of privacy for the Murtaza et al. [17] model is given as:

$$\nabla_{M}=(1-p)[\sigma_{T}^{2}(\sigma_{Y}^{2}+\mu_{Y}^{2})+\alpha^{2}\sigma_{S}^{2}].$$ (29)

The joint measure of privacy and efficiency for the Murtaza et al. [17] model is given as:

$$\delta_{M}=\frac{Var({\hat{\mu }}_{M})}{\nabla_{M}}=\frac{1}{n}[\frac{(1-p)\sigma_{T}^{2}(\sigma_{Y}^{2}+\mu_{Y}^{2})+\sigma_{Y}^{2}+\alpha^{2}\sigma_{S}^{2}}{\left(1-p)\{\sigma_{T}^{2}(\sigma_{Y}^{2}+\mu_{Y}^{2})+\alpha^{2}\sigma_{S}^{2}\right\}}].$$ (30)

The privacy level offered by the Gupta et al. [12] model is given by:

$$\nabla_{G}=(1-A)[\sigma_{T}^{2}(\sigma_{Y}^{2}+\mu_{Y}^{2})]+\sigma_{S}^{2}.$$ (31)

The joint measure of privacy and efficiency for the Gupta et al. [12] model is given as:

$$\delta_{G}=\frac{Var({\hat{\mu }}_{G})}{\nabla_{G}}=\frac{1}{n}[\frac{(1-p)(1-A)\sigma_{T}^{2}(\sigma_{Y}^{2}+\mu_{Y}^{2})+\sigma_{Y}^{2}+(1-p)\sigma_{S}^{2}}{(1-A)\sigma_{T}^{2}(\sigma_{Y}^{2}+\mu_{Y}^{2})+\sigma_{S}^{2}}].$$ (32)

## 4. Efficiency conditions

In this section, the mathematical conditions for the efficiency are derived.

### 4.1 Warner’s [2] model vs. Eichhorn and Hayre [3] model

Warner’s [2] model is more precise compared to the Eichhorn and Hayre [3] model, if

$$Var({\hat{\mu }}_{W})<Var({\hat{\mu }}_{EH}),$$

or

$$\frac{1}{n}(\sigma_{Y}^{2}+\sigma_{S}^{2})<\frac{\sigma_{Y}^{2}}{n}+\frac{\sigma_{T}^{2}(\sigma_{Y}^{2}+\mu_{Y}^{2})}{n},$$

or

$$\sigma_{T}^{2}(\sigma_{Y}^{2}+\mu_{Y}^{2})>\sigma_{S}^{2}.$$ (33)

Condition (33) may not always be true.

### 4.2 Gupta et al. [4] model vs. Bar-Lev et al. [5] model

The Gupta et al. [4] model will be more efficient than the Bar-Lev et al. [5] model, if

$$Var({\hat{\mu }}_{G1})<Var({\hat{\mu }}_{B}),$$

or

$$\frac{1}{n}[\sigma_{Y}^{2}+(1-p)\sigma_{S}^{2})<\frac{1}{n}[\sigma_{Y}^{2}+(1-p)\sigma_{T}^{2}(\sigma_{Y}^{2}+\mu_{Y}^{2})],$$

or

$$\sigma_{T}^{2}(\sigma_{Y}^{2}+\mu_{Y}^{2})>\sigma_{S}^{2}.$$ (34)

Condition (34) is the same as condition (33). This is because the Gupta et al. [4] procedure is the optional variant of the Warner’s [2] model; and the Bar-Lev et al. [5] technique is simply the optional variant of the Eichhorn and Hayre [3] model.

### 4.3 Gupta et al. [12] model vs. Murtaza et al. [17] model

The Gupta et al. [12] model will be more efficient than the Murtaza et al. [17] model, if

$$Var({\hat{\mu }}_{G})<Var({\hat{\mu }}_{M}),$$

or

$$\frac{1}{n}[(1-p)(1-A)\sigma_{T}^{2}(\sigma_{Y}^{2}+\mu_{Y}^{2})+\sigma_{Y}^{2}+(1-p)\sigma_{S}^{2}]<\frac{1}{n}[(1-p)\sigma_{T}^{2}(\sigma_{Y}^{2}+\mu_{Y}^{2})+\sigma_{Y}^{2}+\alpha^{2}\sigma_{S}^{2}],$$

or

$$A(1-p)\sigma_{T}^{2}(\sigma_{Y}^{2}+\mu_{Y}^{2})+\alpha^{2}\sigma_{S}^{2}>(1-p)\sigma_{S}^{2}.$$ (35)

Condition (35) may not always be true.

## 5. Comparison of models

Table 1 presents the variances of the mean under various models for different choices of *α*, and *A*. Tables 2 and 3 show the values of ∇ and *δ*, respectively, for different models.

**Table 1:** Variances of the mean under different models for *μ*_*Y*_ = 10, $\sigma_{Y}^{2}=2$, *n* = 500.

	Model	$\sigma_{S}^{2}=6$, $\sigma_{T}^{2}=8$			$\sigma_{S}^{2}=10$, $\sigma_{T}^{2}=4$		
		*p* = 0.3	*p* = 0.5	*p* = 0.7	*p* = 0.3	*p* = 0.5	*p* = 0.7
*A* = 0.8 *α* = 0.9	Warner [2]	0.016	0.016	0.016	0.024	0.024	0.024
	Eichhorn and Hayre [3]	1.636	1.636	1.636	0.82	0.82	0.82
	Gupta et al. [4]	0.0124	0.01	0.0076	0.018	0.014	0.01
	Bar-Lev et al. [5]	1.1464	0.82	0.4936	0.5752	0.412	0.2488
	Murtaza et al. [17]	1.15612	0.82972	0.50332	0.5914	0.4282	0.265
	Gupta et al. [12]	0.24088	0.1732	0.10552	0.13224	0.0956	0.05896
*A* = 0.5 *α* = 0.6	Warner [2]	0.016	0.016	0.016	0.024	0.024	0.024
	Eichhorn and Hayre [3]	1.636	1.636	1.636	0.82	0.82	0.82
	Gupta et al. [4]	0.0124	0.01	0.0076	0.018	0.014	0.01
	Bar-Lev et al. [5]	1.1464	0.82	0.4936	0.5752	0.412	0.2488
	Murtaza et al. [17]	1.15072	0.82432	0.49792	0.5824	0.4192	0.256
	Gupta et al. [12]	0.5836	0.418	0.2524	0.3036	0.218	0.1324
*A* = 0.2 *α* = 0.3	Warner [2]	0.016	0.016	0.016	0.024	0.024	0.024
	Eichhorn and Hayre [3]	1.636	1.636	1.636	0.82	0.82	0.82
	Gupta et al. [4]	0.0124	0.01	0.0076	0.018	0.014	0.01
	Bar-Lev et al. [5]	1.1464	0.82	0.4936	0.5752	0.412	0.2488
	Murtaza et al. [17]	1.14748	0.82108	0.49468	0.577	0.4138	0.2506
	Gupta et al. [12]	0.92632	0.6628	0.39928	0.47496	0.3404	0.20584

**Table 2:** Values of ∇ under different models for *μ*_*Y*_ = 10, $\sigma_{Y}^{2}=2$, *n* = 500.

	Model	$\sigma_{S}^{2}=6$, $\sigma_{T}^{2}=8$			$\sigma_{S}^{2}=10$, $\sigma_{T}^{2}=4$		
		*p* = 0.3	*p* = 0.5	*p* = 0.7	*p* = 0.3	*p* = 0.5	*p* = 0.7
*A* = 0.8 *α* = 0.9	Warner [2]	6	6	6	10	10	10
	Eichhorn and Hayre [3]	816	816	816	408	408	408
	Gupta et al. [4]	4.2	3	1.8	7	5	3
	Bar-Lev et al. [5]	571.2	408	244.8	285.6	204	122.4
	Murtaza et al. [17]	574.6	410.43	246.26	291.27	208.05	124.83
	Gupta et al. [12]	169.2	169.2	169.2	91.6	91.6	91.6
*A* = 0.5 *α* = 0.6	Warner [2]	6	6	6	10	10	10
	Eichhorn and Hayre [3]	816	816	816	408	408	408
	Gupta et al. [4]	4.2	3	1.8	7	5	3
	Bar-Lev et al. [5]	571.2	408	244.8	285.6	204	122.4
	Murtaza et al. [17]	572.71	409.08	245.45	288.12	205.8	123.48
	Gupta et al. [12]	414	414	414	214	214	214
*A* = 0.2 *α* = 0.3	Warner [2]	6	6	6	10	10	10
	Eichhorn and Hayre [3]	816	816	816	408	408	408
	Gupta et al. [4]	4.2	3	1.8	7	5	3
	Bar-Lev et al. [5]	571.2	408	244.8	285.6	204	122.4
	Murtaza et al. [17]	571.58	408.27	244.96	286.23	204.45	122.67
	Gupta et al. [12]	658.8	658.8	658.8	336.4	336.4	336.4

**Table 3:** Values of *δ* under different models for *μ*_*Y*_ = 10, $\sigma_{Y}^{2}=2$, *n* = 500.

	Model	$\sigma_{S}^{2}=6$, $\sigma_{T}^{2}=8$			$\sigma_{S}^{2}=10$, $\sigma_{T}^{2}=4$		
		*p* = 0.3	*p* = 0.5	*p* = 0.7	*p* = 0.3	*p* = 0.5	*p* = 0.7
*A* = 0.8 *α* = 0.9	Warner [2]	0.002667	0.002667	0.002667	0.0024	0.0024	0.0024
	Eichhorn and Hayre [3]	0.002005	0.002005	0.002005	0.00201	0.00201	0.00201
	Gupta et al. [4]	0.002952	0.003333	0.004222	0.002571	0.0028	0.003333
	Bar-Lev et al. [5]	0.002007	0.00201	0.002016	0.002014	0.00202	0.002033
	Murtaza et al. [17]	0.002012	0.002022	0.002044	0.00203	0.002058	0.002123
	Gupta et al. [12]	0.001424	0.001024	0.000624	0.001444	0.001044	0.000644
*A* = 0.5 *α* = 0.6	Warner [2]	0.002667	0.002667	0.002667	0.0024	0.0024	0.0024
	Eichhorn and Hayre [3]	0.002005	0.002005	0.002005	0.00201	0.00201	0.00201
	Gupta et al. [4]	0.002952	0.003333	0.004222	0.002571	0.0028	0.003333
	Bar-Lev et al. [5]	0.002007	0.00201	0.002016	0.002014	0.00202	0.002033
	Murtaza et al. [17]	0.002009	0.002015	0.002029	0.002021	0.002037	0.002073
	Gupta et al. [12]	0.00141	0.00101	0.00061	0.001419	0.001019	0.000619
*A* = 0.2 *α* = 0.3	Warner [2]	0.002667	0.002667	0.002667	0.0024	0.0024	0.0024
	Eichhorn and Hayre [3]	0.002005	0.002005	0.002005	0.00201	0.00201	0.00201
	Gupta et al. [4]	0.002952	0.003333	0.004222	0.002571	0.0028	0.003333
	Bar-Lev et al. [5]	0.002007	0.00201	0.002016	0.002014	0.00202	0.002033
	Murtaza et al. [17]	0.002008	0.002011	0.002019	0.002016	0.002024	0.002043
	Gupta et al. [12]	0.001406	0.001006	0.000606	0.001412	0.001012	0.000612

## 6. Discussion and conclusion

The current study is based on a neutral comparison of six existing quantitative randomized response models: (i) Warner’s [2] model, (ii) Eichhorn and Hayre [3] model, (iii) Gupta et al. [4] model, (iv) Bar-Lev et al. [5] model, (v) Murtaza et al. [17] model, and (vi) Gupta et al. [12] model. We presented the comparative analysis in a neutral manner, that is, our analysis doesn’t favor one model over the other, it just evaluates the strengths and weaknesses of the models chosen for the comparative analysis.

Table 1 shows that the Gupta et al. [4] model is the most efficient model, whereas the oldest model of Warner’s [2] is the second-best model in terms of efficiency. Moreover, one may also observe that the recently developed model of Murtaza et al. [17] is less precise than the much older model of Bar-Lev et al. [5]. It is also interesting to observe that the newest of the of the six selected models–the Gupta et al. [12] model, is less efficient than the oldest Warner’s [2] model for the selected choices of values of the parameters of scrambling variables. Further, the Murtaza et al. [17] model is also less efficient than the 50 years old Warner’s [2] model.

Model-efficiency is not the only criterion for assessing the quality of a given quantitative randomized response model. The respondents’ privacy-protection offered by the model is also equally important to judge the quality of a randomized response model. The respondent-privacy level can be measured by the value of ∇ with a higher value indicating better level of privacy protection offered by the model. Table 2 displays the values of ∇ for different choices of values of the parameters of scrambling variables. Table 2 indicates that the old Eichhorn and Hayre [3] model has the highest values of ∇, indicating the best level of privacy protection. It is also observed from Table 2 that the Gupta et al. [4] optional model has the smallest values of ∇, making it the worst of all six models.

Finally, as far as the *overall quality* of the six selected models is concerned, the *δ* values are displayed in Table 3. One may clearly observe that the Gupta et al. [12] model has the smallest *δ* values, making it the best among all six models.

We can conclude that a model which performs better on one measure of model-quality may perform worse on other measure. In practice, the researcher may prefer model-efficiency over respondent-privacy, and vice-versa, depending on the requirements of the survey. If model-efficiency alone is preferable, the researcher may choose one model over the other. Likewise, if respondent-privacy level is preferable over efficiency, then another model may be more appropriate. Since the joint measure, *δ*, assigns equal weights to efficiency and privacy, so it alone may not guide the researcher in choosing a particular randomized response model, as efficiency and privacy may not be equally important in practice. Thus, it is recommended to the researchers to keep in mind the particular situation at hand while choosing a randomized response model for data collection on sensitive variables.

## 7. Future research

This paper compares six existing quantitative randomized response models in a neutral manner. There is also a need to conduct a comparative study on qualitative randomized response models. This will help the researchers choose the appropriate model in situations where the variable of interest is of qualitative nature, such as gender, marital status, socio-economic class, etc.
